# Process evaluation of the residential care transition module

**DOI:** 10.1186/s12913-025-13547-2

**Published:** 2025-10-27

**Authors:** Dana P. Urbanski, Robyn W. Birkeland, Elle A. Albers, David L. Roth, Zachary G. Baker, Allison M. Gustavson, Hawking Yam, Joseph E. Gaugler

**Affiliations:** 1 Department of Speech, Language and Hearing Sciences, College of Arts and Sciences, Indiana University Bloomington, Bloomington, IN 47408 USA; 2 Division of Health Policy and Management, School of Public Health, University of Minnesota, Minneapolis, MN 55455 USA; 3 Division of Geriatric Medicine and Gerontology, Johns Hopkins School of Medicine, Johns Hopkins University, Baltimore, MD 21205 USA; 4 Edson College of Nursing and Health Innovation, Arizona State University, Phoenix, AZ 85004 USA; 5 Center for Care Delivery and Outcomes Research, Minneapolis VA Health Care System, Minneapolis, MN 55417 USA; 6 Division of General Internal Medicine, Department of Medicine, University of Minnesota, Minneapolis, MN 55455 USA

**Keywords:** Dementia, Caregiving, Residential long-term care, Support intervention

## Abstract

**Background:**

Dementia caregiver intervention research often lacks focus on mechanisms of benefit. This study addresses this gap through a process evaluation of the Residential Care Transition Module (RCTM), a telehealth intervention designed to assist family caregivers of persons with dementia post-institutionalization, consisting of six sessions with flexible ad hoc support over a 12-month period. This process evaluation describes the RCTM’s content and delivery, examines treatment fidelity, identifies implementation factors that influenced the primary outcomes (caregiver subjective stress and depressive symptoms), and clarifies mechanisms of intervention benefit.

**Methods:**

The RCTM enrolled 240 primary caregivers randomly assigned to the intervention or attention control group (*n* = 120 each). Process/implementation data were collected through study logs documenting session duration, modality, and content, and treatment review checklists completed at four, eight, and 12 months, capturing caregiver ratings of intervention content, utility, and acceptability. Additionally, 30 purposively selected caregivers completed semi-structured interviews probing their perceptions of the intervention. This process evaluation employed a parallel convergent mixed-methods design, integrating quantitative data from longitudinal surveys with qualitative thematic analysis of interviews.

**Results:**

Most caregivers (107; 89%) completed all six intervention sessions; of these, the majority (80; 75%) completed the intervention in four months. Caregivers found the intervention beneficial across multiple domains, expressing strong support for its utility and acceptability. Interviews revealed nine intervention components that facilitated treatment enactment and highlighted mechanisms of benefit. Longitudinal models showed participation in ad hoc intervention sessions was associated with greater benefit over the 12-month period, with higher frequency and longer duration of ad hoc sessions significantly associated with larger reductions in depressive symptoms and care-related stress, respectively.

**Conclusions:**

The RCTM was delivered with high fidelity, demonstrating strong caregiver participation and positive feedback. Qualitative and quantitative data highlight the RCTM’s value in providing emotional support and informational counseling to help caregivers build mastery for managing residential care-related issues. In particular, results demonstrated the potential importance of flexible ad hoc sessions in complementing the core intervention. These insights can inform future adaptation/implementation of the RCTM to support dementia caregivers within residential long-term care settings.

**Trial Registration:**

ClinicalTrials.gov: NCT02915939; 09-26-2016

**Supplementary information:**

The online version contains supplementary material available at 10.1186/s12913-025-13547-2.

## Introduction

Dementia caregiving is associated with a variety of negative health outcomes in family caregivers, including depression, anxiety, and role overload [1]. The challenges of unpaid dementia care (i.e., “caregiving”) are sometimes exacerbated before, during, and after important transitions in the disease, including changes in disease course, healthcare providers, and care settings [2–8]. As a result, caregivers may benefit from the development, evaluation, and dissemination of behavioral interventions tailored to support them at crucial junctures in their dementia care journey [9]. Such interventions should address the complex interplay of caregivers’ evolving informational, educational, and psychosocial needs [10, 11]. Accordingly, *multicomponent interventions*—programs incorporating two or more intervention strategies (e.g., education, training, counseling [12])—may effectively promote caregivers’ well-being while navigating challenges and transitions in dementia care [13, 14].

A well-established literature documents important health benefits of non-pharmacologic, multicomponent interventions for dementia caregivers, including reduced depressive symptoms, decreased burden, and improved subjective well-being [12, 14, 15]. This research has emphasized the results of randomized controlled trials (RCTs), widely considered the “gold standard” for establishing evidence of efficacy/effectiveness. However, dementia care intervention research is limited by insufficient attention to elucidating mechanisms of intervention benefit, identifying target populations, and describing the context and characteristics of intervention delivery. Often, it is unclear which intervention components drive observed benefits and whether some are more beneficial. In short, it is currently unknown why and how most interventions work—and for whom. As a result, practitioners and clinicians are left with limited information to guide the replication of intervention benefits and/or implementation of effective interventions into clinical practice or other real-world settings. Addressing these limitations requires researchers to conduct process evaluations alongside RCTs [16, 17]. To help facilitate the need for process evaluations (analyses that describe intervention implementation and its effect on intervention outcomes), the 2015 Medical Research Council (MRC) process evaluation framework provides valuable guidance for conducting process evaluations to analyze and explain the context, implementation, and mechanisms of impact of complex multicomponent interventions (described in detail later)[18]. 

In this manuscript, we employ the MRC framework to conduct a process evaluation of the Residential Transition Care Module (RCTM), a remotely delivered psychosocial and psychoeducational support program [19, 20]. for family caregivers of individuals with dementia admitted to a residential long-term care (RLTC) setting (i.e., a nursing home or assisted living memory care unit). The RCTM intervention is designed to support caregivers in navigating and adjusting to role changes and psychosocial challenges associated with one of the most significant transitions in the dementia care trajectory—admitting a relative with dementia to RLTC. The efficacy of the RCTM intervention was evaluated in a previously conducted and published randomized controlled trial [20]. Our process evaluation aims to describe the content and delivery of the RCTM intervention, identify process factors that influence intervention outcomes, and clarify mechanisms of intervention benefit.

## The residential care transition module (RCTM)

The RCTM is a six-session semi-structured psychosocial and psychoeducational telehealth intervention designed to assist family caregivers of persons with dementia residing in RLTC facilities, such as nursing homes, memory care, and assisted living memory care units [21]. Admission of a relative to RLTC is a significant transition in the dementia care trajectory, a role which can evoke negative health outcomes in dementia caregivers, who may experience guilt, stress, and depression as they adjust to a new role that is focused less on direct care and more on engagement and advocacy within a care facility [22, 23]. The RCTM intervention supports caregivers through this transition via personalized coaching, information, and resources focused on self-care, stress management, problem-solving, goal-setting, and caregiving skill-building in the RLTC context. Specifically, intervention content is designed to help caregivers remain actively involved in RLTC, advocate for their relative’s care needs, better manage stressors, and interact constructively with their care recipient and residential care staff. More details on the RCTM intervention and evaluation are available below and in the published study protocol [21].

The RCTM intervention and its evaluation were guided by the Stress Process Model for Residential Care (SPM-RC) [24]. Based on the causal assumptions and pathways in the SPM-RC (see below), we hypothesized that the RCTM intervention would significantly reduce subjective stress and depressive symptoms in family caregivers who received the intervention compared to an attention control. The RCTM randomized controlled trial found no significant differences between the treatment and control groups for either quantitative outcome [20]. However, additional analyses indicated that caregivers in the treatment group whose relatives had transitioned into RLTC in the three-month period before joining the study were more likely to report a significant decrease in depressive symptoms during the first four months of the study. This effect dissipated over time and did not reach statistical significance when data from bereaved caregivers were removed [20].

However, qualitative outcome data revealed areas of benefit that were not captured well by quantitative outcomes. In particular, intervention recipients reported meaningful improvements in mood, caregiving confidence, communication, and engagement with care recipients and residential care staff [19]. Participants attributed these benefits to several intervention mechanisms, including education on dementia and behavior management, strategies for improved interactions and communication with care recipients and RLTC staff, personalized resource provision, and emotional support [19]. Given the complex and emotionally charged nature of the transition to residential care—and the mixed results of the randomized controlled trial—this process evaluation was a critical next step to better understand how the RCTM was delivered, how caregivers experienced it, and what unidentified mechanisms of benefit may have contributed to outcomes. Findings from this process evaluation can help identify overarching principles for intervention delivery and mechanisms of benefit that both inform and extend beyond the RCTM itself. By examining the dynamic interplay among implementation, caregiver engagement, and intervention outcomes, this study can guide future adaptation, delivery, and potential scale-up of the RCTM, as well as the development of similar interventions designed to support dementia caregivers during the transition to residential care.

### Process evaluation framework and objectives

This process evaluation was conducted using the MRC process evaluation framework [18], a widely recognized reporting structure for conducting methodologically rigorous process evaluations of complex multicomponent interventions. The MRC framework begins by describing *the intervention and its causal assumptions*, which may be drawn from social science theory and/or clinical experience. The underlying causal assumptions directly shape *implementation*, including how the intervention was delivered (e.g., resources, training), intervention fidelity, and any adaptations made during intervention delivery. Implementation then influences *mechanisms of impact*, which includes the causal pathways through which the intervention brings about change, along with any mediators or moderators of intervention benefit. All aspects of the intervention process are further influenced by the surrounding *context*, broadly defined as anything external to the intervention that might impede or facilitate its delivery and effectiveness. Guided by the above framework, this process evaluation: a) describes the content and delivery of the intervention; b) assesses the intervention’s utility and acceptability; c) evaluates treatment fidelity; d) identifies process factors that influenced intervention outcomes; and e) clarifies mechanisms of intervention benefit.

## Methods

### RCTM randomized controlled trial design

The RCTM evaluation used a single-blind, randomized controlled design. Recruitment and enrollment took place between December 2016 and February 2020. A total of 240 primary caregivers were enrolled, all of whom provided care for a family member diagnosed with Alzheimer’s disease or Alzheimer’s disease-related dementia who was living in an RLTC setting. Eligible caregivers spoke English, were 21 years or older, were not involved in other one-on-one caregiver consultation services, and, if taking psychotropic medications, had been on a stable dosage for at least three months. For more details on recruitment and enrollment procedures, see Gaugler et al. (2020)[21].

The 240 caregivers were from 18 states, representing each geographic region of the United States. The states with the largest enrollments were Minnesota (59%, n = 141) and Wisconsin (12%, n = 28), followed by Ohio (n = 13), New York (n = 12), and California (n = 11), each with about 5% of the sample. The remaining 35 participants (15%) hailed from 13 states. Eleven (9.2%) primary caregivers in the treatment group enrolled with secondary caregivers, nine of whom participated in intervention sessions with the primary caregiver. 10 (8.5%) control group participants enrolled with secondary caregivers.

Following completion of the baseline survey, enrolled caregivers were randomly assigned to the treatment or attention control group and notified by the coordinator of their randomization status. Details about randomization can be found in Gaugler et al. (2024) [20]. Treatment (n = 120) and attention control group participants (n = 117; three caregivers randomized to the control group withdrew from the study immediately following randomization) were assigned to one of the two study coaches after completing the baseline survey. See Table 1 for additional study enrollment information.

**Table 1:** Study enrollment by year

Year	Treatment Enrollees	Control Enrollees	Total Enrollees By Year
2016	8	7^a^	15
2017	35	33^b^	68
2018	44	48	92
2019	30	28	58
2020	3	4	7

*One participant withdrew after randomization. ^b^Two participants withdrew after randomization

Participants in the attention control group could ask for resources throughout their time in the study and were specifically asked if they needed resources from their study coach at four time points: following randomization and during coach-initiated thank-you calls following the completion of the three follow-up surveys. Treatment participants received the RCTM intervention from their study coach, beginning with three weekly sessions followed by three monthly sessions. Additionally, treatment participants were encouraged to participate in ad hoc coaching in addition to their intervention sessions. At any time during their enrollment, treatment participants could choose to engage in individual ad hoc sessions or brief communications, depending on their preference. Ad hoc coaching allowed coaches to address pressing concerns in real time and provide continued support after the intervention was complete. Intervention delivery began in December 2016, with the final session conducted in mid-July 2020.

The RCTM intervention was delivered via phone or secure videoconferencing. Coaches provided emotional support and individualized coaching designed to improve caregivers’ well-being and adjustment to their relative’s transition to RLTC. Two trained coaches (a marriage and family therapist and a PhD in clinical psychology) delivered the intervention. Coaches met weekly and as needed to review participant cases and ensure treatment fidelity. The developer of the RCTM intervention was also available to advise the coaches. Additional information on the coaches’ training can be found in the protocol and main quantitative outcome papers [20, 21].

The second coach (Coach 2) joined the study team in November 2017, completed training under Coach 1, and started delivering the RCTM in January 2018. Generally, caregivers were assigned to their designated coach in alternating order, except when adjustments were made for coach workload. Caregivers participated in the intervention solely with their designated coach. However, at the beginning of April 2020, after completing the intervention with all of their treatment group participants, Coach 1 left the university. At that time, Coach 1’s 29 participants who were still enrolled in the 12-month study (12 treatment, 17 control) were transferred to Coach 2. Coach 2 assumed the coaching role with these participants for the remainder of their time in the study, including offering ad hoc coaching to Coach 1’s remaining treatment group participants.

Participants in both the treatment and attention control groups completed survey measures at baseline, four months, eight months, and 12 months, providing quantitative data about caregivers’ subjective stress, well-being (depressive symptoms), competence and self-efficacy, family involvement, secondary role strain, and stress related to navigating residential care. Treatment participants also completed treatment review checklists (TRCs) at four, eight and 12 months, sharing quantitative data specifically on caregivers’ experiences with the intervention and qualitative feedback from an open-ended item. Semi-structured interviews were conducted with a subset of 30 purposively selected treatment group participants, providing additional open-ended feedback related to participants’ evaluation of the RCTM. Data collection was completed in May 2021. All study procedures were approved by the governing Institutional Review Board, and participants received compensation for their participation.

## Process evaluation design

The RCTM evaluation was guided by the SPM-RC, a conceptual framework that explains how stress impacts family caregivers of individuals with dementia following the transition to RLTC [24]. The SPM-RC is an adaptation of the widely used Stress Process Model (SPM) of dementia caregiving. The SPM is a conceptual model that clarifies the causal pathways through which dementia caregiving influences caregiver well-being over time [25]. According to the SPM, care demands (e.g., activities of daily living dependencies, behavioral challenges, cognitive impairment) act as primary objective stressors, which are then appraised by caregivers as primary subjective stressors (e.g., caregiver role strain, emotional exhaustion). Over time, these care-related stressors accumulate and intensify, spreading to caregivers’ other roles, relationships, and activities. The resulting *proliferation* of stress adversely affects caregivers’ emotional health and overall well-being.

The SPM-RC [24] modifies the SPM by including additional interconnected domains specific to dementia caregiving in RLTC settings. Specifically, the SPM-RC includes domains centered on both the caregiver’s and care recipient’s adjustment to residential care, along with RLTC-specific stressors such as negative interactions and communication challenges between the caregiver and/or care recipient and the residential care staff, other residents, and other families. The SPM-RC emphasizes the contribution of RLTC-specific stressors to caregiver subjective stress and secondary role strain, particularly among caregivers who feel distressed by their care recipient’s placement in RLTC and struggle to form positive relationships with residential care staff. Within this framework, caregivers’ perceptions of their interpersonal relationships with their care recipient, other family members, residential care staff, and other residents and families shape their adjustment to RLTC and overall well-being—as well as their appraisal of their care recipient’s adjustment to RLTC. Following this causal pathway, the RCTM intervention aimed to support caregivers’ adjustment to RLTC by equipping them with strategies for managing stress and improving their interpersonal relationships in RLTC. Accordingly, the RCTM evaluation measured the effect of the RCTM intervention on reducing caregiver subjective stress and depression.

The current process evaluation of the RCTM useods design (QUAN + qual) [26]. Quantitative and qualitative data were analyzed separately and integrated during the interpretation of findings, emphasizing quantitative results with qualitative data providing supporting context and potentially explanatory causal mechanisms for the quantitative results.

## Data collection

### Process/Implementation data

Process data were documented by coaches for all participant encounters (e.g., ad hoc sessions) and included all treatment-related and administrative communication (e.g., session scheduling). Coaches documented participant encounters via Excel tracking logs, recording the session date, duration, modality (email, telephone, videoconferencing, mail, texts), type (coaching, administrative, ad hoc), and broadly, the communication foci. Coaches also completed detailed session notes for intervention sessions and any ad hoc session over 20 minutes. These notes indicated the coach, date, session number, key caregiving issues discussed, family dynamics, caregiver’s reaction to the session, topics to explore in the next session, and coach impressions. Session notes captured dimensions of secondary role strain and caregivers’ experiences of residential care-related stressors, key constructs of in the SPM-RC. Particular attention was paid to examples of tension or communication challenges involving care recipients, family members, and RLTC staff reflecting stress proliferation and relational strain. Session notes and contact logs also served as treatment fidelity indicators.

At four, eight, and 12 months, caregivers provided intervention receipt data via the TRC.

The 21-item TRC assessed the delivery of intervention content focused on stress management and communication skills, informational counseling on dementia and expectations for residential care, and strategies for obtaining support from family and staff, as well as intervention acceptability on a 5-point Likert scale (1 = strongly disagree, 3 = neutral, 5 = strongly agree). Items related to communication and support-seeking were designed to reflect relational strain and caregiver adjustment to RLTC, both central domains in the SPM-RC. An additional open-ended item asked caregivers to share how the counseling had been helpful or any other comments they wished to share about the RCTM (see the Results section below).

Additionally, 30 purposively selected caregivers from the treatment group completed a final semi-structured interview to provide qualitative feedback on their satisfaction with the intervention, highlighting the most valued aspects and its perceived impact on their stress levels and interactions with their care recipient and residential care staff. The interview guide included prompts related to relational strain and adjustment to RLTC, consistent with core constructs from the SPM-RC. Caregivers were selected to ensure a balanced representation based on gender distribution, length of time in RLTC (over and under three months), relationship to the care recipient (adult child or spouse), and satisfaction with the RCTM (average score on the TRC either four and above or under four). Interviews lasted an average of 30 minutes (SD = 12.7; range 15–70 minutes) and followed an interview guide developed for this study (previously published in Albers et al., 2023 [19] and included as supplementary material). Albers and colleagues (2023) [19] detail the characteristics of semi-structured interview participants in the RCTM qualitative analysis.

### Context of care characteristics

Caregivers reported sociodemographic characteristics for themselves and their care recipients. Demographic information included age, gender, race, marital status, education, and employment, along with care recipient-specific information, including dementia severity, type of RLTC setting, and time since RLTC placement. See Table 2 for baseline descriptive characteristics of the study participants.

**Table 2:** Baseline descriptive characteristics (taken from Gaugler et al., 2024 [20])

	Treatment Group (n = 120)	Control Group (n = 120)	Total N	p value
Caregiver Characteristics				
Primary Caregiver N (%)	120 (100.00)	120 (100.00)	240	1.00
Age M ± SD	62.51 ± 10.02	64.00 ± 10.32	229	0.268
Female N (%)	100 (83.33)	100 (83.33)	240	1.00
White N (%)	118 (99.16)	117 (97.50)	239	0.317
Married N (%)	97 (80.83)	95 (79.83)	239	0.846
Earned Bachelor’s degree or higher N (%)	94 (78.33)	90 (75.00)	240	0.542
Employed N (%)	61 (50.83)	43 (35.83)	240	0.019
Income of $40,000 or lower N (%)	14 (12.07)	24 (20.34)	234	0.086
Relationship to Relative N (%)			240	0.312
Spouse	36 (30.00)	40 (33.33)		
Adult Child	81 (67.50)	73 (60.83)		
Other	3 (2.50)	7 (5.83)		
**Relative Characteristics**				
Age M ± SD	83.49 ± 7.99	83.21 ± 9.02	240	0.797
Female N (%)	79 (65.83)	79 (66.39)	239	0.928
White N (%)	114 (95.80)	118 (98.33)	239	0.245
Married N (%)	45 (38.14)	43 (36.13)	237	0.750
Earned Bachelor’s Degree or higher N (%)	45 (38.14)	51 (42.50)	238	0.493
Income of $40,000 or lower N (%)	64 (57.14)	67 (57.26)	229	0.985
Place of Residence N (%)			240	0.916
Nursing home	28 (23.33)	30 (25.00)		
Assisted living - Standard	24 (20.00)	25 (20.83)		
Assisted living - Memory care unit	63 (52.50)	62 (51.67)		
Other	5 (4.17)	3 (2.50)		
Receive Medicaid N (%)	35 (29.66)	37 (30.83)	238	0.844
Diagnosed with dementia N (%)	112 (94.12)	112 (94.12)	238	1.00
Dementia severity				
Time since placement in RLTC (in months) M ± SD	19.49 ± 19.75	18.12 ± 20.55	240	0.599
*Passed away during study period N (%) During intervention N (%)				

Note: M, mean; SD, standard deviation; RLTC, Residential Long-Term Care

### Primary intervention outcome measures

Participants completed measures related to the RCTM’s primary outcomes (subjective stress and depressive symptoms). Primary subjective stress was measured using the seven-item Care Related Strain scale [24], the seven-item Zarit Burden Interview (ZBI [27]), and a single-item measure of the caregiver’s difficulty dealing with their relative’s mental state [24]. Informed by the SPM-RC, the Care Related Strain scale and ZBI were selected to reflect primary subjective stress and stress proliferation, capturing how stress related to caregiving may extend into other domains of function and well-being. Depressive symptoms were measured using two scales: the 15-item Mood Assessment Scale (MAS) [28] and the 20-item Center for Epidemiological Studies - Depression Scale (CES-D) [29]. Once caregivers were bereaved during the study, they received shortened surveys in which the only primary outcomes collected were MAS and CES-D. Details regarding the quantitative measures administered are described in Gaugler et al. (2024) [20].

### Quantitative analysis

As detailed in earlier quantitative analyses of the RCTM [20, 21], a 10% loss to follow-up at each interval across four waves of data were considered when determining statistical power. Enrolling a total of 240 dementia caregivers (*n* = 120 in treatment; *n* = 120 in control) yielded 0.87 power to detect a medium effect size (0.50 standard deviation units). A power of 0.80 was available to detect smaller effects (0.46). The primary outcome measure used to power the analysis was the Zarit Burden Interview (see above). Descriptive analyses were conducted to examine the process indicators of the RCTM intervention. Correlations were also estimated to determine if the baseline primary outcomes were associated with the process indicators. Linear mixed-effects models were then fit to investigate the associations between RCTM process indicators and the primary outcomes over a 12-month period. As in Gaugler et al. (2024) [20], the longitudinal models were centered at four months, and change in the primary outcomes at four, eight, and 12 months was assessed, controlling for the baseline value of the analytic outcome measure. A time-varying binary covariate for caregiver bereavement status was controlled for only in the MAS and CES-D models since these outcomes were assessed at each survey time point for bereaved and nonbereaved caregivers. Nested time points within individuals and an unstructured covariance matrix were specified in the longitudinal models. The models estimated the contrast of the effect of the process indicators at four months (as the models were centered at four months), as well as change over time represented by month effects and interaction effects between month and process indicators.

Individual models were fit for each combination of primary outcome measure and process indicator to examine the association of each process indicator with the primary outcome at four, eight, and 12 months. As mentioned previously, the primary outcome measures included primary subjective stress (PSS) measured using the Care-Related Strain (CRS) Scale, ZBI, and caregiver difficulty dealing with their relative’s mental state using a single-item measure developed by Whitlatch et al [24]. The two other primary outcomes measures were the CES-D and MAS, which both measured caregiver depression. Process indicators included intervention coach identification, TRC mean item score, number of intervention sessions, average length of intervention sessions in minutes, time duration to complete the intervention, participation in ad hoc sessions, total number of ad hoc sessions, and average length of ad hoc sessions. All models were conducted using SAS version 9.4 and *p* < 0.05 was considered statistically significant.

### Qualitative analysis

Qualitative analyses were performed on redacted transcripts from the semi-structured interviews and the TRC open-ended item responses. Interview audio recordings were professionally transcribed and uploaded into NVivo 12 (QSR International Pty Ltd., 2018). We employed Braun and Clarke’s (2006) six steps of thematic content analysis to conduct a reflexive thematic analysis of the transcripts. Using an iterative process, the team created a codebook by reading interview transcripts and generating initial codes. To ensure rigor, reliability, and validity, the team met biweekly to develop the codebook, double code interviews, and resolve coding discrepancies. Categorized codes were grouped to generate preliminary themes and sub-themes. The team then met to discuss the preliminary themes, modifying the codebook as needed to clarify themes, connections, and explanations. Data saturation was achieved when the interviews no longer introduced new content that was not already covered by the codebook (which occurred; please see below). Using the finished codebook, six team members independently coded every transcript and open-ended TRC question response. The team members were divided into pairs to review coding decisions. The codes were then grouped into categories and subsequently into final themes and subthemes. Interrater kappa agreement for interviews was 45%, while percent agreement for TRC open-ended responses was 76% at 4 months, 77% at 8 months, and 81% at 12 months.

## Results

### Intervention utility and acceptability

TRC completion rates were high across time points: four months (*n* = 120), eight months (*n* = 115), and 12 months (*n* = 110). TRCs assessed caregivers’ perceptions of the RCTM’s utility and acceptability. Participants rated each TRC item from 1 (strongly disagree) to 5 (strongly agree), with an option for “not applicable.” At the survey time point immediately following intervention completion, 20 of the 21 TRC items had average scores above 4, indicating that most caregivers believed the RCTM was helpful and increased their understanding of residential care, communication, stress management, relationship dynamics, and their care recipient’s needs (see Table 3 for TRC data). These self-reported improvements reflect key SPM-RC domains, including primary subjective stress, interpersonal tensions, and adjustment to residential long-term care. Caregivers’ high retention and completion rates reflected their positive perceptions of the RCTM. Treatment group participants found the RCTM highly acceptable, with the strong endorsement of items such as recommending the RCTM to others in similar situations (*M* = 4.64) and to those whose relatives were experiencing similar circumstances (*M* = 4.66). Scores for 20 of the 21 items increased between the 4-month and 12-month surveys. The only item that did not increase, “Become aware of services and supports outside of my relative’s facility,” decreased slightly from 4.14 to 4.08, still reflecting high agreement with the statement.

**Table 3:** Treatment review checklist overall averages

Average Item Scores for First Survey Completed after Intervention			
The counseling sessions helped me:	Coach 1	Coach 2	Across Coaches
1. Understand how admitting a relative to residential care affects each person in your family.	4.18 SD = 0.95	4.39 SD = 1.00	4. 26 SD = 0.96
2. Understand how care is provided to your relative in her/his residential care setting.	3.94 SD = 1.11	4.36 SD = 1.01	4.11 SD = 1.09
3. How memory care for your relative can be optimized in residential long-term care.	3.93 SD = 1.09	4.43 SD = 0.83	4.13 SD = 1.02
4. Express my needs to other family members.	4.17 SD = 0.92	4.29 SD = 1.01	4.23 SD = 0.95
5. Understand my family member’s needs.	4.23 SD = 0.84	4.36 SD = 0.90	4.29 SD = 0.86
6. Understand my relative’s needs.	4.24 SD = 0.95	4.43 SD = 0.93	4.32 SD = 0.94
7. Establish more positive relationships with facility staff.	4.10 SD = 0.98	4.36 SD = 0.99	4.21 SD = 0.99
8. Break up problems with my relative into manageable pieces.	4.17 SD = 0.97	4.24 SD = 0.93	4.20 SD = 0.95
9. Become aware of services and supports in my relative’s facility.	3.92 SD = 0.95	4.20 SD = 0.94	4.03 SD = 0.96
10. Become aware of services and supports outside of my relative’s facility.	4.07 SD = 0.95	4.24 SD = 0.80	4.14 SD = 0.89
11. Understand how my changing role because of my relative’s placement was a potential source of stress.	4.50 SD = 0.97	4.75 SD = 0.60	4.60 SD = 0.85
12. Deal with my relative’s behaviors in the residential care facility.	4.21 SD = 1.00	4.52 SD = 0.71	4.33 SD = 0.90
13. Communicate with my relative more effectively.	4.25 SD = 0.92	4.45 SD = 0.83	4.34 SD = 0.89
14. Communicate with staff more effectively in caring for my relative.	4.18 SD = 1.01	4.52 SD = 0.82	4.32 SD = 0.95
15. Enhance support from family members and friends.	4.01 SD = 0.90	4.13 SD = 0.93	4.05 SD = 0.91
16. Develop care plans with other family members and/or facility staff.	4.12 SD = 0.98	4.13 SD = 0.92	4.12 SD = 0.95
17. Establish goals for my relative’s care.	4.10 SD = 0.93	4.26 SD = 0.93	4.17 SD = 0.93
18. Understand my relative’s memory loss and/or dementia	4.28 SD = 1.02	4.67 SD = 0.72	4.44 SD = 0.93
19. Understand the available treatments and their effectiveness for my relative’s memory loss and dementia.	3.71 SD = 1.07	4.07 SD = 1.00	3.84 SD = 1.05
20. I would recommend the Residential Care Transition Module to others in a similar situation as my relative is.	4.61 SD = 0.81	4.69 SD = 0.78	4.64 SD = 0.79
21. I would recommend the Residential Care Transition Module to others in a similar situation as I am.	4.63 SD = 0.80	4.71 SD = 0.74	4.66 SD = 0.78
Average of Averages	4.17	4.39	4.26

## Treatment fidelity

The TRCs, study tracking logs, and semi-structured interviews served as indicators of treatment fidelity. These sources of qualitative and quantitative data, when compared, demonstrated convergence that the intervention was delivered as intended with high fidelity, that participants received and understood the treatment, and that participants enacted treatment skills that led to perceived benefits [30]. These findings are described in detail below.

### Delivery of treatment: implementation, type, and furation of RCTM coaching

Most participants (107; 89%) had six intervention sessions, except for 13 whose care recipients passed away during intervention delivery. After a care recipient’s passing, the intervention was discontinued, and participants were given the option to continue meeting with their coach for ad hoc support. Nine of the 13 bereaved participants continued with ad hoc coaching. These participants engaged in an average of four post-bereavement sessions (SD = 3.7) with an average session length of 49 minutes, SD = 24.4 minutes). Of the 107 participants who completed the intervention, 80 (75%) completed the intervention in four months, while the remaining completed the intervention in five or more months.

`Almost all treatment group caregivers (*n* = 114, 95%) engaged in at least one ad hoc session or ad hoc communication during or following the six RCTM sessions. A total of 516 ad hoc sessions were conducted. Caregivers who engaged in ad hoc sessions typically completed multiple sessions, averaging 6 ad hoc sessions (range: 1–38 sessions, SD = 5.7). The majority of intervention and ad hoc sessions were conducted via telephone. Only ten caregivers (8.3%) utilized secure videoconferencing for their intervention sessions, while just 7 (6%) utilized it for ad hoc coaching sessions. Email communication was commonly utilized as a means of ad hoc support. Eighty-one treatment group caregivers (68%) emailed their coach for ad hoc support, including 23 participants who solely utilized email communication to engage in ad hoc coaching. There were 1032 instances of written ad hoc communication, including 972 emails between participants and coaches, 43 text messages between eight participants and their coach (most text exchanges consisted of notification that a relative had passed and the coach offering support), and 15 mailings of resources to participants. Table 4 displays intervention, ad hoc session, and control group resource provision data by coach.

**Table 4:** Intervention and ad hoc sessions

		Coach 1	Coach 2	Across Coaches
Treatment	Caregivers Assigned	72 (3*)	48 (8*)	120
	Total Number of Sessions	407	279	686
	Average Session Length (Minutes)	76 Range 18–125 SD = 22.6	87 Range 55–130 SD = 18.3	81 Range 18–130 SD = 21
Ad hoc	Caregivers	44 (61%)	37 (77%)	81 (68%)
	Average Number of *Ad Ho*c Sessions	4 Range 1–12 SD = 3.5	8 Range 1–23 SD = 7.2	6 Range 1–23 SD = 5.7
	Average Session Length (Minutes)	43 Range 15–70 SD = 18.5	39 Range 14–86 SD = 17.9	41 Range 14–86 SD = 18.2
Control	Caregivers Assigned	63 (9*)	54 (4*)	117

*Number of secondary caregivers completing surveys

Note: One of Coach 1s secondary caregivers participated in the intervention sessions. All of Coach 2s secondary caregivers participated in intervention sessions. Of note, five treatment group participants received ad hoc coaching from Coach 2. These five caregivers participated in an average of 6 ad hoc sessions with Coach 2, an average session length of 58 minutes

As intended, attention control group participants did not receive routine ad hoc consultation. However, when asked if they would like resources during their quarterly thank-you calls, 34 (29%) control group participants asked for and received resources (12 [24%] of Coach 1 and 22 [40%] of Coach 2’s control group participants). This ad hoc contact ranged from one to eight resource-related emails or calls over the study period (*M* = 1.97, SD = 1.4). The duration of supportive contact with attention control participants via phone and email ranged from five to 35 minutes (*M* = 15 minutes, SD = 8.20).

Coaches communicated via email with participants for various administrative reasons, most commonly for scheduling purposes (treatment participants) and, as noted above, resource provision (attention control participants). These administrative emails were typically brief, with 99% of them written or read by the coach in five to ten minutes. Table 5 provides detailed information on these administrative coach-caregiver email exchanges.

**Table 5:** Administrative email communication

	Coach 1	Coach 2	Across Coaches
Total Number of Emails between Coaches and Participants	n = 455 *C*^*a*^*initiated* n = 270 *CG*^*b*^*initiated* n = 185	n = 816 *C initiated* n = 522 *CG initiated* n = 294	n = 1271 *C initiated* n = 792 *CG initiated* n = 479
Average Duration (minutes)	5.78 Range 5–30 SD = 2.8	5.09 Range 5–45 SD = 1.5	5.31 Range 5–45 SD = 2.0
Treatment Group Only (minutes)	*C initiated* n = 261 5.38 Range 5–10 SD = 1.34 *CG initiated* n = 182 6.35 Range 5–30 SD = 4.07	*C initiated* n = 474 5.03 Range 5–10 SD = 0.40 *CG initiated* n = 288 5.01 Range 5–10 SD = 0.29	*C initiated* n = 735 5.16 Range 5–10 SD = 0.87 *CG initiated* n = 470 5.53 Range 5–30 SD = 2.62
Control Group Only (minutes)	*C initiated* n = 9 5.56 Range 5–10 SD = 1.67 *CG initiated* n = 3 8.33 Range 5–10 SD = 2.89	*C initiated* n = 48 6.06 Range 5–45 SD = 5.89 *CG initiated* n = 6 5.0 Range 5–5 SD = 0.0	*C initiated* n = 57 5.96 Range 5–45 SD = 5.38 *CG initiated* n = 9 6.11 Range 5–10

^a^C = coach, ^b^CG = caregiver

Study tracking logs showed that intervention session content aligned closely with the RCTM design and causal assumptions. Records showed that most intervention sessions centered on providing emotional support. Coaches also delivered psychoeducation, encouraged positive communication, taught communication skills and strategies, and engaged the participant in active problem-solving, often amid discussions regarding the relative’s behavior or care issues involving the RLTC facility. These topics—particularly emotional support, family dynamics, and staff-related issues—align with the SPM-RCs emphasis on interpersonal challenges and subjective stress following RLTC transitions. Ad hoc sessions/communications also most often focused on emotional support. Since ad hoc sessions were used to address immediate issues, they frequently centered on problem-solving, RLTC care concerns, and promoting constructive communication with the care recipient, staff, or other family members. Participants reported crises during ad hoc communication more often than in regular intervention sessions. The specific foci of intervention sessions (*n* = 686) and all ad hoc communications (*n* = 1548), including written exchanges, are detailed in Table 6.

**Table 6:** Intervention content foci

Content Foci	Intervention Sessions with Content Focus n(%)	Ad Hoc Session/Communications with Content Focus n(%)
Emotional Support	617 (91)	820 (53)
Relative’s Behavior	534 (78)	379 (24)
Promotion of Communication	511 (75)	410 (26)
Problem-Solving	504 (74)	451 (29)
Facility Issues	469 (69)	518 (33)
Psychoeducation	412 (60)	401 (26)
Concrete Services/Referral	40 (6)	139 (9)
Making Families Aware of Treatment	30 (4)	54 (3.5)
Crisis	1 (0.14)	43 (3)

### Receipt of treatment

The TRC included several items that assessed participants’ understanding of the material covered during RCTM sessions. Results from the TRC (see Table 3 for item averages) indicated that caregivers felt the intervention improved their understanding of how residential care impacts other family members, how care is provided in RLTC, how to communicate their needs to other family members, and how to recognize both their family’s and care recipient’s needs. Additionally, caregivers reported that the intervention helped them understand how to build better relationships with RLTC staff, break down care-related problems for more effective problem-solving, manage care recipient behaviors in RLTC, communicate more effectively in RLTC, and set care goals. As previously noted, TRC item averages reflecting participants’ understanding of the intervention material were near ceiling, except for one item that asked whether the RCTM increased participants’ “understanding of available treatments and their effectiveness for my relative’s memory loss and dementia treatments” (*M* = 3.84; SD = 1.05). Together, TRC results are consistent with positive perceptions of intervention benefit in key SPM-RC domains of secondary role strain and adjustment to changing caregiving responsibilities and relational contexts within RLTC.

Coach-recorded session notes indicated that participants actively demonstrated their understanding of the intervention material during sessions. Many detailed their plans for applying communication strategies from the RCTM to improve their interactions and care coordination with family members and residential care staff. Participants also worked with coaches to identify essential points to address at their next care conference and practiced discussing these topics during sessions. In line with the RCTM’s emphasis on self-care, participants frequently expressed intentions to use relaxation exercises introduced and practiced during their intervention sessions. Additionally, many participants collaborated with coaches to develop personalized engagement activities to try with their relatives during upcoming visits.

### Enactment of treatment skills and mechanisms of benefit

Qualitative analyses of the 30 semi-structured interviews and an open-ended item on the follow-up TRCs identified nine intervention components that facilitated the enactment of treatment skills and illuminated mechanisms of intervention benefit, demonstrating convergence with the available quantitative treatment fidelity data. These mechanisms stem from two key aspects of the intervention: 1) education (information and strategies); and 2) support from the coach. The mechanisms are described below. All participant names are pseudonyms. These pseudonyms are not linked to participants’ real names or identifying information, and were assigned to protect confidentiality.

Education: Information and Strategies. Caregivers found significant value in six content areas related to education: information and strategies (for additional detail, see Albers et al., 2023 [19]).

Dementia Education. Caregivers valued learning about dementia progression and the management of behaviors [19]. They felt their knowledge impacted how they responded to their care recipient. They were less stressed, calmer, and more patient, reflecting the RCTM’s emphasis on reducing emotional reactivity and stress proliferation. Sarah (daughter, interview) shared, “It lowered the stress and the frustration I was feeling, so I could help my Dad feel better with the frustration and things he was dealing with because I was so calm and more understanding and knowledgeable about what was going on.” Sharon (daughter, interview) noted, “I was more patient with her. Learning more about the disease. It was like, okay, this is what’s going on. This is how I need to react.” Similarly, Tammy (daughter, interview) stated, “I think because of what I have gained through [RCTM], I think I am better equipped to be with her, and not escalate behaviors.”

Ideas for Activities and Engagement. Caregivers appreciated hearing about activities and ways to engage with their care recipient [19]. Tony (husband, interview) shared that he now knows “a lot more than what I knew before, on how to keep her calm and things to do with her, things to try to keep her mind occupied.” Mary (daughter, interview) shared how the RCTM impacted her ability to engage with her mother:

[Coach] and I would brainstorm how I can do stuff. Taking stuff to share with her … and using that as a way of sparking conversations, not trying to draw her back into my reality, but allowing her to enjoy something in the moment so that it gave us something to focus on. So, learning how to just sort of be in the moment with her really helped, and we spent a lot of time, Coach and I, talking about how I might be able to engage her in a way that isn’t threatening so that Mom wouldn’t panic.

Communication Strategies. Participants also benefited from communication strategies to use with their care recipient, family, and healthcare team [19]. Mary (daughter, interview) shared how she found it helpful learning “ways of talking to [CR] and learning how to talk with her so that she doesn’t feel like she’s not a whole person,” adding that “recognizing that she doesn’t remember it and always having that in the back of my mind has helped me tremendously in being able to stay calm with her.” Betsy agreed, “I think some of the ideas about how to communicate with her…. I just think learning those things improves my ability to interact with my mom and have a good experience.” Sarah (daughter, interview) shared how the RCTM impacted her communications with RLTC personnel, “I felt like I was able to communicate better with the staff and was more confident being able to ask the questions that I did to the staff.” Amanda (daughter-in-law, interview) noted, “[Coach] really empowered me … helping me better prepare to talk to the staff, and really get a good [care] conference … than I would have if I had just done it by myself.” Improved communication within caregiving and RLTC systems suggests the RCTM helped reduce relational tensions and supported adaptation to RLTC, consistent with SPM-RC mechanisms.

Relaxation Techniques. Caregivers also highlighted the usefulness of learning relaxation strategies, which helped reduce the immediate impact of caregiving and residential care-related stressors and may have prevented their proliferation, consistent with the SPM-RC [19]. Andi (daughter, 8-month TRC), who participated in the intervention with her sister, explained that the relaxation techniques impacted both their self-care and their care recipient’s well-being, “the exercises that we got to help us relax help not only us, but our mom too. Lily (daughter, 4-month TRC) expressed how the RCTM decreased her caregiver stress and improved her self-care, “Since counseling with [Coach], I have been able to reduce my stress level re: caring for my Dad. I am better at caring for myself.”

Provision of Outside Resources. Caregivers also appreciated receiving resources that coaches tailored to their needs and priorities [19]. When asked to name topics that were more impactful, Kendra (daughter, interview) responded, “The ones where we talked about resources available,” later specifically adding that RCTM helped relieve some of her stress and burden because it “geared me to some resources.” Rachel (daughter, 12-month TRC) also valued the resources her coach found for her, “I was very glad for the support I received from the counseling sessions. I was given helpful suggestions of online resources, books, and ideas each time.” Charlotte (daughter, 12 months TRC) shared how the resources she received helped her, her family, and her friends, “I was promptly provided with any requested resources/additional reading … It’s been a real asset to use the knowledge gained to support my Dad and other relatives … I’ve also been able to share what I’m learning with friends who have relatives with dementia.”

Management of Responsibilities. Caregivers recognized the benefit of exploring how to manage multiple roles to better manage residential-care related stressors [19]. Tara (daughter, interview) shared about navigating her full-time job and caregiving duties, “I was still coping with caring for my mother, and it was not always so easy in juggling my various responsibilities…we talked about that, and I found that was useful and helpful in providing support.” Caroline (daughter, 8-month TRC) clarified her roles within her family, “It has been extremely helpful for me to understand my role not only as a caregiver and daughter but also to understand my relationships with my siblings.”

Transition Coach Support. Caregivers found three elements of coaching support the most impactful: emotional support, knowledge, and being a neutral third party [19]. Amanda (daughter-in-law, interview) summarized the benefit of all three elements of coaching support, sharing appreciation for having “someone I could vent to, that really had more knowledge than just a random friend would have, and more sympathy and understanding.”

Emotional Support and Reassurance. Caregivers most valued that coaches were understanding, reassuring, and supportive [19]. Sharon (daughter, interview) emphasized the reassurance she felt working with her coach, “And I think that’s the key thing, just reassurance. Yes, you are doing the right thing. But here are some other things that may help.” Eva (daughter, 8-month TRC) felt sessions with her coach “reaffirmed what I was doing and were confidence builders.” Coaches were also supportive by validating caregiver concerns and sharing that other caregivers faced similar caregiving experiences. This support engendered a sense of community for caregivers who often felt alone. As Lisa (niece, 4-month TRC) shared, “I feel less alone … I feel like the path we are on is not unusual and that we, too, can get through this as others have. It’s amazing to feel like I have a support system.”

Knowledgeability. Caregivers also appreciated that coaches were knowledgeable about dementia and the adjustment to having a relative live in an RLTC setting [19]. Jack (son, interview) felt confident: “Because I had someone supporting me, she was knowledgeable, and I knew I could ask her questions about different things.” Brianna (daughter, 4-month TRC) concurred, “Having someone knowledgeable about dementia as an individual resource was very valuable.”

Neutral Third Party. Caregivers expressed great comfort in talking openly to a neutral third party who did not judge them and whom they did not burden [19]. Brianna (daughter, 4-month TRC) found talking to “someone who could empathize and not judge … was very comforting.” Ben (son, interview) stated, “I think the big thing was it’s nice to have somebody to talk to that is more objective…I knew that [Coach] would ask questions and we could have discussions … but in the larger community, I don’t really probably as much as maybe I could or should,” Jillian (wife, 8-month TRC) shared how she benefited from talking with an objective third party, “Just being heard clearly by someone who was not herself overwhelmed by what has happened in the lives of my husband and I, gave me a different perspective.”

## Empirical associations between process indicators and key outcomes

As mentioned previously, most participants completed the full six-session intervention in four months. Due to the lack of variability in both the number of intervention sessions and the time duration to complete the intervention, these process indicators were not analyzed further. The remaining process indicators were included for further analysis: coach identification, TRC mean item score, average length of intervention sessions in minutes, participation in ad hoc sessions, total number of ad hoc sessions, and average length of ad hoc sessions.

Prior to running statistical models, correlations were estimated to analyze associations between baseline primary outcomes and the process indicators of interest. Most correlations between process indicators and primary outcomes were not statistically significant. However, several correlations were statistically significant: ZBI and participation in ad hoc sessions (*r* = 0.22, *p* = 0.014), CES-D and total number of ad hoc sessions (*r* = 0.27, *p* = 0.013), CRS and the average length of ad hoc sessions (*r* = 0.26, *p* = 0.021), and ZBI and average length of ad hoc sessions (*r* = 0.25, *p* = 0.025).

Longitudinal linear mixed-effects modeling showed that the number of ad hoc sessions and the average time spent per ad hoc session were associated with intervention benefit, as indicated by significant month-by-process indicator interaction effects (Table 7). Table 7 displays the results of all statistical models conducted for this analysis. Statistically significant findings are discussed below. All other estimates were not statistically significant.

**Table 7:** Longitudinal mixed models effects of RCTM process indicators on primary analytic outcomes

	Month Effect		RCTM Process Indicator Effect		Month ×RCTM Process Indicator Effect	
	Estimate (CI)	*p* value	Estimate (CI)	*p* value	Estimate (CI)	*p* value
Average Length of Intervention Sessions (in minutes)						
CRS	−0.05 (−0.46, 0.36)	0.808	0.02 (−0.02, 0.06)	0.375	−4.30e^−4^ (−0.01, 0.01)	0.863
ZBI	0.05 (−0.31, 0.42)	0.777	0.02 (−0.02, 0.05)	0.354	−2.14e^−3^ (−0.01, 2.20e^−3^)	0.329
Mental State	−7.53e^−3^ (−0.07, 0.06)	0.811	2.35e^−4^ (−4.03e^−3^, 0.01)	0.913	−4.38e^−6^ (−7.50e^−4^, 7.42e^−4^)	0.991
CES-D*	0.45 (−0.38, 1.28)	0.281	0.06 (−0.01, 0.12)	0.076	−8.59e^−3^ (−0.02, 1.24e^−3^)	0.086
MAS*	0.03 (−0.17, 0.22)	0.804	8.94e^−3^ (−0.01, 0.03)	0.275	−6.00e^−4^ (−2.94e^−3^, 1.73e^−3^)	0.611
Participated in Ad Hoc Sessions (Yes/No)						
CRS	−0.16 (−0.34, 0.02)	0.077	0.56 (−1.19, 2.30)	0.528	0.11 (−0.11, 0.33)	0.306
ZBI	−0.16 (−0.31, 6.62e^−4^)	0.051	0.11 (−1.37, 1.59)	0.880	0.05 (−0.14, 0.24)	0.602
Mental State	−0.03 (−0.06, −2.48e^−3^)	0.032	−0.10 (−0.29, 0.08)	0.274	0.03 (8.00e^−4^, 0.06)	0.056
CES-D*	−0.42 (−0.79, −0.05)	0.026	−1.66 (−4.56, 1.23)	0.257	0.25 (−0.19, 0.69)	0.263
MAS*	−0.07 (−0.16, 0.02)	0.123	0.11 (−0.61, 0.82)	0.766	0.07 (−0.04, 0.17)	0.222
Number of Ad Hoc Sessions						
CRS	0.07 (−0.11, 0.25)	0.430	0.11 (−0.05, 0.28)	0.175	−0.02 (−0.04, 2.08e^−3^)	0.077
ZBI	−0.09 (−0.26, 0.09)	0.323	0.09 (−0.05, 0.24)	0.213	−3.52e^−3^ (−0.02, 0.02)	0.730
Mental State	0.01 (−0.02, 0.03)	0.609	1.32e^−3^ (−0.02, 0.02)	0.885	−5.20e^−4^ (−3.27e^−3^, 2.23e^−3^)	0.708
CES-D*	0.19 (−0.19, 0.57)	0.327	0.43 (0.15, 0.72)	0.003	−0.06 (−0.10, −0.01)	0.013
MAS*	0.08 (−6.89e^−3^, 0.17)	0.070	0.12 (0.05, 0.19)	0.001	−0.02 (−0.03, −4.42e^−3^)	0.006
Average Length of Ad Hoc Sessions (in minutes)						
CRS	0.29 (−0.03, 0.60)	0.075	−3.68e^−3^ (−0.06, 0.05)	0.895	−7.60e^−3^ (−0.01, 8.20e^−4^)	0.029
ZBI	−4.83e^−3^ (−0.32, 0.31)	0.976	−0.02 (−0.07, 0.03)	0.484	−2.31e^−3^ (−0.01, 4.41e^−3^)	0.494
Mental State	0.01 (−0.03, 0.06)	0.536	−1.26e^−3^ (−0.01, 4.68e^−3^)	0.672	−2.30e^−4^ (−1.13e^−3^, 6.61e^−4^)	0.603
CES-D*	0.01 (−0.67, 0.70)	0.973	0.05 (−0.05, 0.14)	0.321	−4.02e^−3^ (−0.02, 0.01)	0.594
MAS*	−0.09 (−0.25, 0.07)	0.269	−0.01 (−0.04, 0.01)	0.238	2.06e^−3^ (−1.46e^−3^, 5.57e^−3^)	0.248
TRC Mean Score						
CRS	0.31 (−0.45, 1.07)	0.422	−0.10 (−1.42, 1.22)	0.877	−0.09 (−0.27, 0.08)	0.304
ZBI	−0.05 (−0.73, 0.63)	0.885	−0.27 (−1.37, 0.84)	0.635	−0.02 (−0.17, 0.14)	0.833
Mental State	0.01 (−0.11, 0.12)	0.874	−0.12 (−0.26, 0.02)	0.094	−3.85e^−3^ (−0.03, 0.02)	0.773
CES-D*	−1.13 (−2.40, 0.13)	0.079	−1.54 (−3.47, 0.39)	0.116	0.21 (−0.09, 0.50)	0.164
MAS*	−0.12 (−0.42, 0.18)	0.434	−0.08 (−0.57, 0.40)	0.732	0.02 (−0.05, 0.09)	0.534
Coach ID						
CRS	−0.08 (−0.21, 0.06)	0.266	0.87 (−0.78, 2.52)	0.297	−0.02 (−0.23, 0.19)	0.837
ZBI	−0.11 (−0.23, 0.01)	0.065	0.26 (−1.13, 1.65)	0.711	−0.02 (−0.20, 0.16)	0.832
Mental State	−3.90e^−3^ (−0.02, 0.02)	0.705	−0.11 (−0.29, 0.07)	0.237	−8.50e^−3^ (−0.04, 0.02)	0.591
CES-D*	−0.20 (−0.48, 0.07)	0.145	1.86 (−0.86, 4.57)	0.178	−0.12 (−0.53, 0.30)	0.586
MAS*	−0.01 (−0.07, 0.06)	0.860	0.27 (−0.40, 0.94)	0.429	−0.04 (−0.14, 0.05)	0.367

Note: RCMT, Residential Care Transition Module; CI, Confidence Interval; CRS, Care-Related Strain; ZBI, Zarit Burden Inventory; CES-D, Center for Epidemiological Studies - Depression Scale; MAS, Mood Assessment Scale; TRC, Treatment Review Checklists

*Models include bereaved and nonbereaved caregivers, unlike the other models

Dementia caregivers who participated in higher numbers of ad hoc sessions demonstrated improvements in several primary outcomes over the one-year intervention period, controlling for baseline values of the analytic outcome measure. For each additional ad hoc session attended, caregivers’ MAS scores were 0.12 higher at four months compared to caregivers who requested no ad hoc sessions during the 12-month study (SE = 0.04, *p* < 0.01). However, for each ad hoc session caregivers attended, their MAS scores were −0.02 lower each month (SE = 5.26e^−3^, *p* < 0.01) compared to caregivers who requested no ad hoc sessions – indicating a reduction in depressive symptoms associated with the use of ad hoc sessions. The same trend was observed for CES-D, wherein for each ad hoc session attended, caregivers’ CES-D scores were 0.43 higher at four months compared to caregivers who had no ad hoc sessions during the study (SE = 0.14, *p* < 0.01). For each ad hoc session attended, caregivers’ CES-D scores decreased by −0.06 each month (SE = 0.02, *p* < 0.05) compared to caregivers who had no ad hoc sessions.

The average length of ad hoc sessions was significantly associated with decreased care-related strain over time. For each additional minute in ad hoc sessions, care-related strain decreased slightly throughout the study compared to caregivers who had no ad hoc sessions (B = −7.60e^−3^, SE = 3.38e^−3^, *p* < 0.05).

## Discussion

The convergence of the quantitative and qualitative data available in the RCTM process evaluation indicate that this multicomponent intervention—designed to alleviate stress during and after the institutionalization of a cognitively impaired relative—was well received by participants and delivered with a high degree of fidelity. Participants endorsed near-ceiling ratings of the RCTM and showed strong adherence to the formal intervention. In keeping with the formal intervention schedule, dementia caregivers participated in the six counseling sessions within the first four months and engaged in ad hoc sessions as needed. The primary mode of delivery of the RCTM was via telephone, although use of email was common for ad hoc support. It is important to note that most of the primary counseling sessions of the RCTM were delivered prior to the onset of the COVID-19 pandemic; it is possible due to increasing use of videoconferencing during the pandemic by older adults and their caregivers [31, 32], use of this mode of delivery will be more acceptable to dementia caregivers in current and future interventions (for COVID-19 related findings from the RCTM, please see Mitchell et al., 2021 [33] and Mitchell et al., 2023 [34]).

Intervention session logs and qualitative interview data highlighted the importance of emotional support as the most frequently addressed need during RCTM counseling and ad hoc sessions, often due to challenges/concerns related to behaviors on the part of cognitively impaired relatives in RLTC settings or staff interactions/care quality issues. Perhaps due to their structure, ad hoc sessions often focused on immediate problem-solving of issues or sometimes crises that emerged for dementia caregivers often related to behaviors on the part of relatives in RLTC or staff-related communication challenges.

The empirical data collected from treatment review checklists (TRCs) highlighted the RCTM’s acceptability and feasibility, as well as the potential ways dementia caregivers utilized the intervention’s consultation to address RLTC-related issues and concerns. Immediately after the delivery of the RCTM counseling component, dementia caregivers were highly likely to recommend the intervention to those in similar situations, and also indicated that the RCTM counseling helped caregivers to understand their changing role following a relative’s admission to RLTC. Among other areas where the RCTM appeared to provide use and benefit for dementia caregivers were understanding needs of cognitively impaired care recipients and family members, addressing dementia-related behaviors, and improving communication. Overall, participants in the RCTM rated the utility and benefits of the intervention highly on the TRCs administered throughout the course of the 12-month evaluation.

Longitudinal analysis of process variables on primary RCTM outcomes yielded several key findings. Given the lack of variability in duration and frequency of use of formal counseling sessions, it is perhaps unsurprising that these process variables had few statistically significant associations with primary RCTM outcomes. However, given the greater variability in use and length of time in ad hoc sessions, several statistically significant interactions emerged. Participants who used ad hoc counseling sessions more frequently or for longer periods of time on average per session were more likely to indicate statistically significant decreases in stress or depressive symptoms, controlling for their baseline levels. These findings could suggest the presence of a booster effect for the RCTM, where ongoing engagement with counselors following the six-session, 4-month intervention period alleviated distress over time and potentially limited stress proliferation – in line with the predictions of the SPM-RC. However, given the observational nature of these associations and limited control for selection bias, alternative explanations remain plausible. Future research should directly test the hypothesis that participant-initiated ad hoc coaching provides additional benefit beyond the structured RCTM intervention.

A particular benefit of integrating qualitative data into the overall RCTM trial was that such information indicated mechanisms of action/benefit that drove positive outcomes on the part of intervention participants. One mechanism was the provision of specific strategies that improved management, support, and engagement. Learning about strategies to effectively engage with care recipients, manage multiple care roles, identify external resources, communicate, and practice relaxation techniques were also aspects of the RCTM that seemed associated with greater benefit on the part of dementia caregivers. These domains align with the underlying mechanisms of the SPM-RC, which emphasize the importance of addressing primary caregiving stressors and those specific to RLTC in order to prevent stress proliferation and related declines in caregivers’ functioning and well-being. A second mechanism of action was perceptions of support from RCTM coaches. Coaches in the RCTM were viewed as sources of direct emotional support as well as “neutral third parties” that helped offer unbiased information to dementia caregivers while navigating RLTC for their relatives. The embedded qualitative data of the RCTM suggested that these mechanisms of action appeared to drive reductions in stress and depressive symptoms among dementia caregivers, and yielded additional insights as to how the intervention/coaching process itself was potentially beneficial to RCTM participants.

The original conceptual framework of the RCTM relied on the Stress Process Model, tailored for residential long-term care (SPM-RC) [24, 25]. The use of embedded qualitative data suggested that the RCTM effectively targeted several of the resource domains of the SPM-RC (e.g., emotional support, information/education to build mastery related to various care-related issues) to potentially influence key outcomes on the part of dementia caregivers. In addition to these mechanisms, structural aspects of the RCTM appeared to empirically drive benefits for dementia caregivers: specifically, the length and duration of ad hoc sessions following the standard six sessions of the RCTM coaching modules. However, the use of a robust process evaluation of the RCTM offers stronger insights into the “form”and “function” of the RCTM. Core functions indicate how the components of intervention activities operate to influence outcomes (akin to mechanisms) “… why what is being done is important, and how activities work to ultimately change behavior, structures, processes and/or outcomes” [35] P 328 If shown to be effective, the core functions of an intervention should not be changed/dismissed as part of any adaptation process [35]. Alternatively, “forms” are activities of the intervention: who is doing what, when, where, and how in a given intervention (for the RCTM, transition coaches provided six individual psychosocial and psychoeducational support sessions within 4 months, with ad hoc/ongoing consultation provided up to an additional 8 months delivered over the telephone or secure videoconferencing. A stated goal in intervention design is to map forms to core functions, ideally a priori; however, as in the current mixed methods process evaluation, the qualitative data offered a more compelling, in-depth picture of those core functions and how they are “mapped” onto the RCTM coaching content following trial completion. As a next step in RCTM intervention development, we plan to identify whether existing quantitative measures of the mechanisms highlighted here and in our other qualitative RCTM research [19] were measured either at the item- or scale-level and to ascertain whether the RCTM intervention through its psychosocial and psychoeducational content influenced: a) mechanisms of action identified in the qualitative RCTM data; and b) benefits/outcomes also specified in the open-ended data and semi-structured interviews collected from treatment participants.

There are several important limitations to note. The RCTM sample was not diverse ethnically or racially and was English-speaking, despite our efforts to engage RLTC providers nationally to result in a more representative sample. Compared to national caregiver profiles from the National Study of Caregiving and Alzheimer’s Association data, the RCTM caregiver sample had a higher proportion of women and White caregivers, and overrepresented spouses and adult children as primary caregivers, with fewer caregivers in other family or friend roles [36–39]. However, unlike national samples that include multiple caregivers and caregivers for a range of health conditions, our sample was limited to primary caregivers of individuals with dementia, which may partially account for these differences. Nevertheless, caution is warranted when generalizing these findings to other caregiver populations. Importantly, the representativeness/generalizability of the sample is further hindered due to its voluntary nature.

Future testing and adaptation of the RCTM must include diverse caregivers to more deeply examine how both individual-level characteristics (e.g., cultural beliefs about caregiving, help-seeking behaviors, communication preferences, religious beliefs) and context-level factors (e.g., access to technology, availability of culturally and linguistically concordant providers, and systemic inequities in residential care settings) may influence engagement with the intervention, perceived support, and resulting outcomes. These dimensions could meaningfully shape both how the intervention is delivered (form) and how it achieves its effects (function) across diverse populations. In such cases, cultural adaptation and intervention co-creation strategies may be necessary to modify RCTM content, delivery methods, and/or outcome measures to better reflect variations in caregiving behavior and beliefs among racially, ethnically, and linguistically diverse caregivers. Efforts to scale the RCTM will depend on this work, particularly given the limited representativeness and generalizability of the current study sample.

Several other limitations should be noted. A more concerted effort to collect information at the facility level may have further informed the process evaluation findings presented here. The RCTM intervention’s target/focus is on primary caregivers, which may not reflect the more dynamic, multi-family member aspects of dementia caregiving. Given the multiple statistical comparisons of this process evaluation, statistically significant findings should be interpreted with caution due to potential Type I errors.

Although still fairly uncommon, process evaluations can help to inform the translational research process. As was evident in the RCTM, treatment adherence and delivery of this telehealth intervention demonstrated high fidelity, which suggests that the intervention has potential scalability for other organizations to implement (although an implementation concern not considered in our RCTM efficacy trial is the degree of professional expertise necessary to deliver the RCTM coaching sessions). The use of multiple methods in our process evaluation also demonstrated that, in addition to a possible “booster” effect when using ad hoc counseling sessions, a number of mechanisms appear to drive potential benefits of the RCTM. These essential aspects of the intervention can guide adaptation efforts if the RCTM is implemented in additional RLTC settings to support dementia caregivers following a relative’s institutionalization.

## Electronic supplementary material

Below is the link to the electronic supplementary material.

Supplementary Material 1

## Data Availability

The data, analytic methods, and study materials are available at https://doi.org/10.3886/E198382V1. This study is registered at https://clinicaltrials.gov/ (NCT02915939).

## Abbreviations

RLTC: Residential Long-Term Care
RCTM: Residential Care Transition Module
MRC: Medical Research Council
SPM: Stress Process Model
SPM-RC: Stress Process Model for Residential Care
TRC: Treatment Review Checklist
CRS: Care-Related Strain
MAS: Mood Assessment Scale
CES-D: Center for Epidemiological Studies - Depression Scale
ZBI: Zarit Burden Inventory
