# Supplementary material for: Process evaluation of the residential care transition module
Source: BMC Health Serv Res. 2025 Oct 27;25:1412. doi: 10.1186/s12913-025-13547-2 (PMC12560283; doi:10.1186/s12913-025-13547-2)
Supplement: Supplementary file 1 — Supplementary Material 1 [file 12913_2025_13547_MOESM1_ESM.docx]

**Appendix A.** The Residential Care Transition Module Interview Questions Guide

Phase I: Background of CRs memory loss and CG living status

1: Can you tell me a little bit more about CR and how you first found out about their memory problems?

*Probes:*

*a.* *How long ago was it that you made the decision to place CR in a long-term care facility?*

*b.* *How are things going now for you?*

Phase II: Overview of RCTM

2: Why did you decide to enroll in this project and to use the RCTM?

3: What was your overall impression of the program?

*Probes:*

*a.* *Do you feel like there were any negative outcomes that resulted from your use of the counseling program?*

*b.* *Any negative outcomes for CR from your using it?*

*c.* *Were there any benefits that you saw, for you in particular, from using the RCTM?*

*d.* *Do you think there were any benefits for your father from your using the RCTM?*

Phase III: Service components of the RCTM and outcomes

4: I'd just like to walk through some of the service components of the RCTM program and then ask how each component either did or didn't help you and CR, and why. First, are there any individual counseling sessions or topics that you felt were best? Anything that jumps out at you that way?

*Probes:*

*a.* *Are there any individual counseling sessions or topics that you felt were best?*

*b.* *Were there any sessions or any topics that you felt were least helpful?*

*c.* *How did discussing your father's care with TC help, or not help?*

5: I'd like to ask you about some links to potential outcomes. Do you feel like your use of the RCTM program had any effect on your feelings of stress and burden?

*Probes:*

*a.* *Do you feel like your use of the RCTM had any effect on your interactions with CR?*

*b.* *Do you feel like your use of the RCTM had any effect on your interactions with the care staff at the long-term facility?*

*c.* *Did your use of the RCTM have any effect on your interactions with your other family members?*

6: The next outcome I want to ask you about is your ability to stay involved with CR's care.

*Probes:*

*a.* *Do you feel like your use of the RCTM had any effect on your ability to engage with and stay involved with his care at the facility?*

*b.* *Do you feel like your use of the RCTM had any effect on your feelings of being down or being blue?*
